# Unilateral Hearing Loss and Dental Care: A Questionnaire Survey of Patient Challenges and Provider Responses

**DOI:** 10.1111/scd.70264

**Published:** 2026-09-21

**Authors:** Jumpei Murakami, Yumi Okano, Yuki Oda

**Affiliations:** ^1^ Department of Special Needs Dentistry Graduate School of Dentistry The University of Osaka Suita Osaka Japan; ^2^ Department of Speech‐Language‐Hearing Therapy Faculty of Rehabilitation Gunma Paz University Takasaki Gunma Japan; ^3^ Hiroshima Oral Health Center Hiroshima Japan

**Keywords:** communication support, dental accessibility, environmental adjustments, patient‐centered care, unilateral hearing loss

## Abstract

**Aim:**

Unilateral hearing loss (UHL) refers to significant hearing impairment in one ear, with normal or near‐normal hearing in the other. Individuals with UHL often have difficulty hearing in noisy environments, such as dental offices. This study was undertaken to examine the specific needs of individuals with UHL related to the dental care delivery process, such as speaking from the hearing side and reducing background noise.

**Methods:**

A web‐based survey was conducted through the official website of Kikoiro, a Japanese community organization focused on individuals with UHL. Participants were individuals with UHL, defined as normal hearing in one ear and hearing loss of 25 dB HL to complete deafness in the other ear. Those with bilateral hearing loss and family members or proxies were excluded. The purpose, target population, and important information regarding the survey were clearly stated on the website. A link to the survey form, created using the SurveyMonkey application, was posted, with responses collected in a voluntary manner.

**Results:**

A total of 378 individuals with UHL participated in the survey. Many reported difficulties communicating in a noisy dental environment. Necessary adaptations noted included clear and slow speech, reduced background noise, visual cues, and communication from the hearing side, while visual aids and written explanations before and after treatment were also considered helpful. The results indicate the importance of specific communication and environmental adjustments when providing dental care for this population.

**Conclusions:**

In dental clinic settings, consideration of patient hearing ability is essential, especially for those with UHL. Improving the communication environment benefits not only affected patients but also contributes to more inclusive dental care.

## 1 Introduction

Unilateral hearing loss (UHL), defined as normal hearing in one ear and a significant hearing impairment in the other, affects approximately 7.9% to 13.3% of the general population [1, 2]. Prevalence varies among age groups, with studies conducted in the United States noting that from 0.3 to 1.0 per 1000 newborns [3, 4, 5], 0.5% to 5% of school‐aged children [6], and up to 7.2% of older adults [7] were affected.

Although individuals with UHL generally perform well in a quiet environment, they often face substantial communication difficulties in noisy settings [8]. Welsh et al. presented findings showing that monosyllable recognition in a setting with background noise significantly declines in individuals affected by UHL [9]. Moreover, another study showed that brain activity patterns differ depending on whether the right or left ear is affected by hearing loss [8], suggesting that the side of impairment can influence auditory performance, with different accommodations required.

Results of a speech perception experiment conducted to compare children with UHL and those with normal hearing found that the latter group had accuracy of 99% in a quiet condition and of 90% in a noisy condition, with the decline not significant [10]. In contrast, they found that those with UHL exhibited a significant drop in hearing accuracy from 95% to 84% in a noisy environment (*p* < 0.05). These findings indicate specific challenges in real‐world listening environments faced by children with UHL.

A dental clinic environment typically includes high levels of background noise from such equipment as suction devices and high‐speed drills, with levels near the face of patients found to reach 86–96.7 dB [11]. Such conditions pose particular challenges for individuals with UHL, who rely heavily on their ear with good hearing, and may be unable to effectively localize a sound or clearly hear what is important when background noise is too great [12]. Unfortunately, UHL is often overlooked in clinical settings as the patient can hear well on one side. As a result, they might not receive appropriate accommodation and staff members may lack awareness regarding their specific communication needs, which can lead to misunderstanding, dissatisfaction with the treatment given, or even avoidance of dental care [13].

There is growing recognition that hearing accessibility is not only relevant for patients who are deaf or with bilateral hearing problems, but also for those with UHL. However, few studies have explored the effects of UHL on the dental care experience or what environmental factor adjustments patients find helpful.

This study was undertaken to clarify the challenges faced by individuals with UHL during dental care and to identify the types of adjustments that may improve accessibility. Understanding these needs is essential for developing more inclusive and effective dental care practices for patients with unilateral hearing impairment.

## 2 Materials and Methods

### 2.1 Study design

A descriptive cross‐sectional questionnaire‐based study was conducted using a web‐based survey distributed through the official website of Kikoiro, a patient‐led community for individuals with UHL in Japan. The associated webpage included clear explanations regarding the survey purpose, eligibility criteria, and instructions. A formal sample size calculation was not performed because no prior studies were available to provide the parameters required for estimating the expected effect size. Therefore, all eligible respondents during the survey period were included in this exploratory study.

### 2.2 Participants

Individuals interested in participating accessed the online questionnaire using a link provided by SurveyMonkey (San Mateo, CA, USA) and provided responses in a voluntary manner. The survey targeted individuals with UHL, and responses by family members or proxies were not accepted. Data were collected from participants with UHL who provided informed consent and completed the questionnaire between September 1 and November 30, 2023. For the present study, diagnostic criteria for UHL were having normal hearing in one ear and hearing impairment ranging from 25 dB HL to complete deafness in the other ear, while the inclusion criterion was having UHL. Individuals with bilateral hearing loss, and family members or proxies were excluded. This study was approved by the Ethics Committee of the Graduate School of Dentistry and the University Dental Hospital at the University of Osaka (approval No. R5‐E5), and performed according to the ethical guidelines of the Declaration of Helsinki.

### 2.3 Questionnaire

The questionnaire was developed in a collaborative manner by special care dentists and speech‐language‐hearing therapists. Required completion time was estimated to be approximately 10 min. Initially, the participants were asked to confirm their consent to participate in the study and verify that they were individuals with UHL. The questionnaire items were developed based on recurring concerns and communication challenges reported by individuals with UHL during routine clinical encounters. Although a formal pilot study was not conducted, these clinical insights were used to ensure the clarity, relevance, and appropriateness of the questionnaire content for the target population. The questionnaire was then presented in the following five sections.

(1) Demographic information (age, sex, laterality of hearing loss, degree of hearing loss, onset of condition).

(2) Dental visits (experiences with visits, timing of most recent visit, regularity of dental visits, disclosure of UHL to dental clinic staff).

(3) Specific difficulties encountered in dental settings (before visit, upon arrival, in examination room, during treatment, after treatment, after visit).

(4) Preferred accommodations in dental settings (overall staff, reception and waiting area, treatment room, post‐treatment reception).

(5) Three open‐ended questions regarding positive and negative experiences with dental care, and suggestions for improving the environment were also included, with the answers provided by each participant in a text format.

### 2.4 Data Analysis

Statistical analyses were performed using IBM SPSS Statistics, version 29. For qualitative analysis of open‐ended responses, two independent researchers separately performed inductive open coding to identify emerging themes and then the findings were compared. Substantial agreement was achieved between them, indicating a high degree of consistency in thematic interpretation. Discrepancies were collaboratively reviewed and resolved through discussion until consensus was obtained. Based on this process, a finalized coding framework was developed and applied to the full dataset. Quantitative data were analyzed using descriptive statistics. Fisher's exact test was employed for between‐group comparisons involving categorical variables due to unequal variances and skewed distributions. A *p*‐value < 0.05 was considered to indicate statistical significance. No adjustment for multiple comparisons was made due to the exploratory nature of the study.

## 3 Results

### 3.1 Participant Characteristics (**Table** **1**)

A total of 378 individuals who stated that they were affected by UHL participated in the survey, most of whom were from 20 to 59 years old, with the majority female (72%). The side of hearing loss was nearly evenly distributed. As for degree of hearing loss in one ear, 44% reported profound deafness, followed by 34% with severe to profound loss. In terms of onset, 25% had congenital hearing loss, while 35% developed that during childhood and the remainder after reaching adulthood.

### 3.2 Dental Clinic Visit Experience (**Figure** **1**)

The vast majority of participants (*n* = 367, 97%) reported prior experience with dental visits and only two indicated no such experience, while nine did not reply to that question. Regarding their most recent visit, that was noted to have occurred by 217 within the past 6 months, by 42 within 6 months to 1 year, by 60 within 1–3 years, by 24 within 3–5 years, and by 24 more than 5 years ago. Concerning regularity of dental care, 253 (66.9%) answered that they attended dental appointments on a regular basis (e.g., every few months, every six months, or annually), whereas 113 (29.9%) did not receive such routine dental care. When asked whether they had disclosed their UHL condition to dental staff, only 135 (35.7%) answered affirmatively, while most participants (*n* = 233, 59.0%) had not informed the clinic staff regarding their hearing disability.

### 3.3 Specific Difficulties Encountered in Dental Settings (**Table** **2**)

Prior to the visit, many noted a sense of anxiety due to concerns about potential communication problems (38.9%) or tension because of their hearing difficulties (33.6%). While in the waiting room, 42.1% felt anxious before the examination. When in the examination room, more than half reported difficulty hearing dental hygienists or assistants (56.4%), or the explanations provided by the dentist (52.1%). Notably, 45% of respondents noted pretending to hear and 42.6% that they used their imagination to interpret spoken information. Environmental noise was also a barrier, especially in the period following the COVID‐19 pandemic (20.1%). During treatment, 43.7% could not hear instructions given by the dentist. After treatment, 30.2% felt uncomfortable while waiting for billing due to uncertainty about whether their name was being called. Additionally, 22.0% reported a sense of fatigue following the visit, and 9.0% felt unsafe outside of the clinic due to difficulty with detection of approaching vehicles or bicycles.

### 3.4 Preferred Settings in Dental Clinic (**Table** **3**)

Regarding overall staff‐related communication, the most commonly preferred approach was clear speaking, selected by 55.8% of respondents, which was followed by reducing surrounding noise as much as possible when speaking (46.0%), speaking loudly (42.6%), and speaking slowly (32.8%). In the reception and waiting areas, 58.2% of the participants preferred a visual display of call numbers to help them know when they were being called. In the treatment room, the most frequently noted request was for clinicians to be aware of and speak to the hearing side of the patient (68.3%).

### 3.5 Responses and Suggestions Related to Positive and Negative Experiences (**Table** **4**)

In addition to the structured survey responses, text responses from the participants regarding their dental clinic experiences were analyzed and categorized into three groups; difficulties experienced (*n* = 133), positive experiences (*n* = 75), and suggestions for improvement (*n* = 115). The most frequently reported difficulty was the auditory environment (e.g., background music, equipment noise, being spoken to on the impaired side), followed by insufficient understanding or support from staff. Positive experiences included being spoken to on the side with better hearing, use of visual aids, and clear speech. The participants also noted preference for a quieter environment, consideration of easy ways to communicate in light of their hearing loss, and facility/system improvements, such as adjustable chairs and visual name/number calling systems. These qualitative findings complemented the structured responses, and highlighted the diverse challenges and needs of individuals with UHL in dental care contexts.

### 3.6 Differences in Dental Difficulties and Requested Adjustment Based on Side of Hearing Loss (**Table** **5**)

A significantly greater number of participants with right‐side hearing loss reported difficulties in multiple situations, particularly when listening to dental clinic assistants positioned on their right. For example, they were more likely to report difficulty hearing the voice of the dentist (65.5% vs. 34.6%, *P* < 0.001), though fewer had difficulties with understanding instructions from an assistant (17.7% vs. 41.2%, *P* < 0.001). Additionally, those with right‐side UHL more frequently expressed a desire for face‐to‐face communication before undergoing treatment (*p* = 0.001).

## 4 Discussion

The present study is among the first to investigate specific difficulties faced by individuals with UHL encountered during dental care and accommodations they consider to be helpful. Although UHL is often regarded as less disabling than bilateral hearing loss [14], the present individuals with UHL noted substantial barriers encountered in dental clinic settings, including communication challenges and anxiety, which often led to avoidance of care.

Despite these challenges, the needs of individuals with UHL are frequently overlooked in clinical practice. This may stem from the misconception that one normal‐hearing ear is sufficient for effective communication, particularly in a quiet environment [15]. As a result, patients with UHL are often not considered for disability support systems and healthcare providers may not be aware of their specific communication needs. Notably, dental clinics are often noisy with a fast‐paced environment, in which patients are reclined for examinations and treatments, the clinicians wear a mask, and visual cues are often limited. Under such conditions, UHL can be a significant barrier to safe and effective care.

A key finding of the present study is that nearly half (45%) of the participants reported pretending to hear during dental consultations, a behavior that likely reflects lack of a sense of psychological safety, as the affected patient may fear that they are burdening staff, misunderstood, or perceived as difficult. While this coping strategy may help them adapt in the short term, it increases the risk of miscommunication, treatment errors, and patient dissatisfaction. The present findings are in agreement with prior research that highlighted communication barriers as a major obstacle to dental care for patients with hearing impairment [16, 17].

Even though 97% of the present participants noted a prior dental clinic visit experience and over two‐thirds that they received dental care on a regular basis, only 35.7% had disclosed their hearing difficulty to staff at the clinic. This low rate suggests that many individuals with UHL desire to avoid bringing attention to their condition; possibly due to fear of stigma, lack of trust that others will understand, or assumptions that helpful accommodations are unavailable or not considered necessary [18, 19]. Consequently, their needs remain unrecognized during treatment, which may contribute to increased anxiety and dissatisfaction with the care provided.

Findings obtained from analysis of responses to the open‐ended questions provided important details related to patient experiences and indicated the depth of the challenges faced by individuals affected by UHL. Several of the respondents described heightened anxiety and fatigue due to uncertainty regarding the clinic procedures. One wrote, “I'm always on edge during a first‐time visit to a dental clinic because I don't know when or how I'll be called. It's exhausting.” Another commented, “Since I can't hear with my right ear and the dentist is always on that side, I get anxious and often can't catch what they're saying.” It was also noted that environmental factors such as machine noise and use of a face mask exacerbated these communication difficulties, based on such comments as, “I couldn't hear the instructions over the suction noise,” and “When the dentist speaks through a mask, I can't see their mouth and I miss what they say.”

Positive experiences were also described and showed how small intentional actions can have a significant effect. For example, one participant noted, “The dentist taps my shoulder before speaking and always makes sure to face the side I can hear from. It makes me feel safe and respected.” Another also explained, “Written explanations and visual cues help me understand what to expect, even when I have missed what was said aloud.” These narratives highlight the impact of empathetic multimodal communication.

The participants also expressed strong preferences for specific communication strategies and environmental modifications. Clear speech, speaking to the hearing side of an affected patient, and a reduction in ambient noise were the most often cited needs. Additionally, visual displays, such as a numbered call system or written explanations, were also highly valued. Such accommodations are relatively easy to implement and have minimal cost, though can greatly enhance patient comfort, accessibility, and satisfaction.

It is important to note that hearing loss laterality was found to influence patient experiences. Participants with right‐sided UHL were more likely to have difficulty understanding clinicians, particularly when they stood on the left side. Such a spatial mismatch can be corrected by the dental staff making simple positional adjustments. Neurophysiological study results indicate that the right ear provides more direct input to the language‐dominant left hemisphere [20, 21], thus right‐sided hearing loss may have more pronounced effects on verbal communication.

Interestingly, the COVID‐19 pandemic has also emerged as a contextual factor with influence on communication. The widespread use of face masks as well as physical distancing hindered access to facial expressions and lip‐reading cues, which are important compensatory points for people with hearing impairment [22]. As for those affected by UHL, such pandemic‐related changes significantly heightened communication barriers, thus highlighting the need for more visual and flexible communication practices in healthcare environments.

A notable strength of this study is the large and diverse group of individuals with UHL recruited from a patient‐led community network, which facilitated collection of rich real‐world insights. Another strong point is the detailed qualitative analysis performed. Open‐ended responses were independently coded by two researchers and substantial agreement was achieved, thus enhancing the reliability and credibility of the findings. Discrepancies were resolved through discussion to reach consensus, with a finalized coding framework developed to support thematic interpretation.

On the other hand, this study also has several limitations. First, all analyzed data were self‐reported, thus there is potential for recall bias and subjective interpretation. Second, participant recruitment was conducted with an online patient community, possibly resulting in selection bias that favored individuals who were more literate, engaged, or advocacy oriented in regard to healthcare issues, which may limit the generalizability of the findings to all individuals affected by UHL. Finally, the cross‐sectional design precludes any inference of causality between hearing characteristics and dental care experiences. Future related studies should incorporate objective audiometric data, expand the recruitment strategy to include individuals who are not often online, and provide means for dental care providers to provide their perspective. Longitudinal or interventional studies would also be valuable for assessing the effectiveness of targeted adjustments aimed at improving care for individuals with UHL.

Findings obtained in the present study highlight challenges faced by individuals with UHL when seeking dental care that are often overlooked. Additionally, they demonstrate that UHL is not a minor condition, but one that requires thoughtful and context‐specific modifications, especially for acoustic and visually complex clinical environments. To promote psychological safety, raising provider awareness and implementing practical adjustments can provide more inclusive patient‐centered dental care that is accessible for individuals with hearing difficulties.

### 4.1 Limitations

The wide age range of participants may have influenced the accuracy and consistency of responses. Differences in cognitive ability, comprehension, and literacy across age groups could have affected how the questions were understood and answered. In addition, proxy responses may have occurred among younger participants or those with limited ability to complete the questionnaire independently. These factors should be taken into account when interpreting the findings.

## 5 Conclusions

This study identified specific challenges experienced by individuals with UHL during dental care, particularly those related to communication and the clinical environment. Participants reported that difficulties in hearing, understanding spoken information, and maintaining comfort were common during dental visits. The study also clarified the types of accommodations that individuals with UHL perceive as helpful, including speaking from the better‐hearing side, using visual supports, and minimizing background noise.

These findings highlight the need for dental professionals to recognize UHL as a distinct hearing disability and to incorporate simple, practical adjustments into routine practice. Implementing such strategies may enhance communication, reduce stress, and promote a more inclusive and accessible dental care environment for patients with UHL.

## Conflicts of Interest

The authors declare no conflicts of interest.

6

**FIGURE 1:**
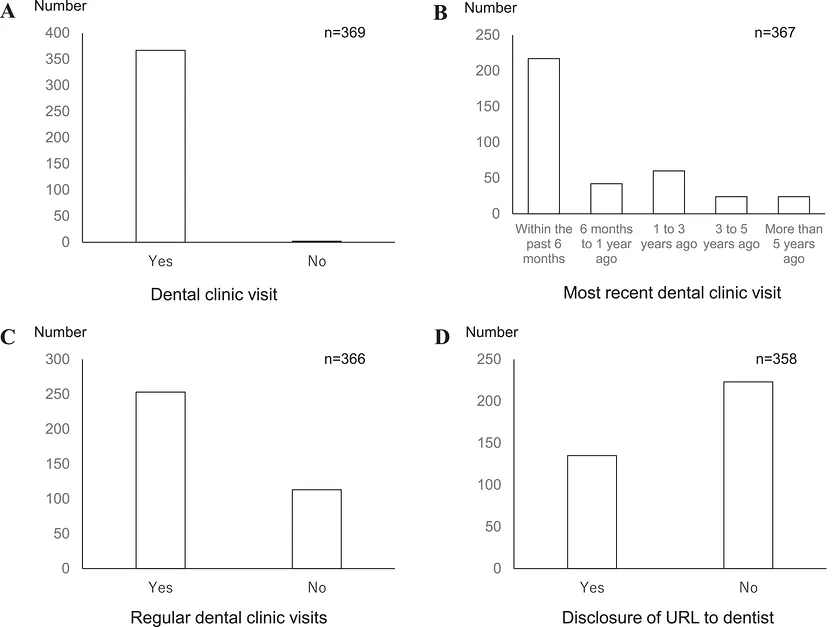
Participant responses regarding dental care experiences and hearing status. (A) Dental clinic visit. (B) Most recent dental clinic visit. (C) Regular dental clinic visits. (D) Disclosure of UHL to dentist.

**TABLE 1:** Demographic information.

	Category	No.	%
Age (years)	< 10	6	1.6
	10–19	12	3.2
	20–29	63	17
	30–39	56	15
	40–49	92	24
	50–59	97	26
	60–69	39	10
	70–79	6	1.6
	≥ 80	2	0.53
	N/A	5	1.3
Sex	Female	274	72
	Male	92	24
	Declined to answer	7	1.9
	N/A	5	1.3
Laterality of hearing loss	Right	220	58
	Left	153	40
	N/A	5	1.3
Degree of hearing loss	Mild to moderate	67	18
	Severe to profound	130	34
	Profound deafness	165	44
	Unknown	11	2.9
	N/A	5	1.3
Onset of hearing loss	Congenital	95	25
	Childhood	131	35
	Adulthood (> 3 years ago)	102	27
	Adulthood (≤ 3 years ago)	45	12
	N/A	5	1.3

N/A: No Answer.

**TABLE 2:** Difficulties experienced by individuals with UHL in dental settings.

Situation	Difficulties	No.	%
Before visit	Worried about potential problems due to hearing issues	147	38.9
	Tension due to hearing difficulty	127	33.6
	Anxious about loud noises near the ears	40	10.6
	Did not want to visit clinic because of hearing issues	28	7.41
	None	134	35.5
Upon arrival (before consultation)	Felt tense while waiting for call due to hearing difficulty	159	42.1
	Had trouble hearing call to enter clinic	135	35.7
	Disturbed by ambient noise in waiting room	35	9.26
	Could not clearly hear sound of TV	18	4.76
	None	130	34.3
In examination room (other than during treatment)	Had difficulty hearing dental hygienists and assistants	213	56.4
	Had difficulty hearing dentist explanations and voice	197	52.1
	Pretended to hear*	170	45.0
	Interpreted spoken content through imagination	161	42.6
	Environmental noise (i.e., vacuum, air purifier) became louder after COVID‐19, making hearing difficult	76	20.1
	Unsure when to listen carefully	50	13.2
	Became reluctant to visit due to dislike of repeated inquiries	26	6.88
	Felt that results were different from expected due to misunderstandings	18	4.76
	Felt uncomfortable when asked to repeat	12	3.17
	Gave up visiting because of feeling burdensome to others by asking again	7	1.85
	Staff members skipped explanations when hearing difficulties expressed	5	1.32
	None	62	16.4
During treatment (in examination room)	Could not hear the dentist's instructions	165	43.7
	Felt even more tense due to difficulty hearing	118	31.2
	Could not hear instructions of assistant when on right side	102	27.0
	Focused on listening when device sounds stopped	79	20.9
	Did not notice the dentist's voice	68	18.0
	Felt discomfort with sound of equipment	62	16.4
	Did not notice assistant's instructions (usually when on the right side)	60	15.9
	Felt discomfort when being examined and explained simultaneously	22	5.82
	Refrained from speaking out despite anxiety or pain to avoid interrupting treatment	18	4.76
	None	65	17.2
After treatment	Could not relax while waiting for billing paperwork, unsure when called	114	30.2
	Had difficulty hearing post‐treatment instructions	78	20.6
	Had to ask for clarification during billing	78	20.6
	Missed the call for billing paperwork	43	11.4
	Had difficulty understanding medication instructions	27	7.14
	Missed call and was moved to back of queue	3	0.79
	None	133	35.2
After visit	Always felt fatigued after dental visit	83	22.0
	Felt unsafe in parking area due to unawareness of approaching cars or bicycles	34	8.99
	Could not perform daily tasks after visit	7	1.85
	None	209	55.3

**TABLE 3:** Preferred adjustments in dental settings noted by individuals with UHL.

Category	Adjustment	No.	%
Overall staff	Speak clearly	211	55.8%
	Reduce surrounding noise as much as possible when speaking	174	46.0%
	Speak loudly	161	42.6%
	Speak slowly	124	32.8%
	Use models or pictures when explaining	90	23.8%
	Make mouth visible while speaking	56	14.8%
	Write down essential contents of conversation on paper	41	10.9%
Reception and waiting area	Display call numbers	220	58.2%
	Use pager (portable device handed out at reception that notifies turn through display screen or vibration)	110	29.1%
	Display subtitles on TV	48	12.7%
Treatment room	Speak from side where hearing is possible	258	68.3%
	Treat in a quiet environment	149	39.4%
	Explain treatment procedure before starting	111	29.4%
	Use visual aids (pictures, photos, diagrams, models, etc.) while explaining	99	26.2%
	Treat in a private room	91	24.1%
	When instructing to open mouth during treatment, use gestures as well as voice	76	20.1%
	Sit upright and face patient before speaking	65	17.2%
	Instead of explaining while looking at or touching inside of the mouth during treatment, explain immediately afterward	51	13.5%
Post‐treatment reception	When billing, verbally inform amount due, an also display it with text or on screen	175	46.3%
	Explain medication while showing prescription	109	28.8%

**TABLE 4:** Summary of text responses to open ended questions regarding dental care experiences and needs of individuals with UHL.

Category	Details	No. of responses
**A. Difficult experience (*n* = 133)**		
Acoustic environment	Voice from affected side, inaudible due to machine noise or BGM, masked mouth	66
Lack of understanding	No information shared, requests ignored	18
Difficulty in understanding	Cannot identify speaker, unable to respond during treatment, unaware of being spoken to	11
Ear symptoms other than hearing loss	Tinnitus, dizziness, auditory hypersensitivity	7
Effort required for listening	Tension at initial visit, forced posture, fatigued by extended concentration	6
Hesitation or reluctance	Hesitant to ask for support or repeat	6
Manner of speaking	Spoken to loudly, voice too soft	3
Use of hearing devices	Must remove hearing aid, concern about device damage	3
No problems	No problems due to acquired coping strategies or proper support	12
Others	Concern about impact on hearing side	1
**B. Positive experience (*n* = 75)**		
Direction of speech	Speech from better‐hearing side	13
Consideration for clarity	Signals, visual support, easy‐to‐understand explanations	13
Manner of speaking	Slow and clear speaking	11
Awareness of hearing loss	Staff aware of hearing loss	11
Quiet environment	Private rooms, no background music, quiet environment	10
Timing of speech	Spoken to calmly, not during treatment	6
Ease of asking for repetition	Atmosphere allowed for easy clarification	6
Advance explanation	Explanations about sounds and procedures	2
Others	Considerations for call‐in and physical condition	3
**C. Desired improvements (*n* = 115)**		
Sound environment adjustment	Quiet environment, machine off, private room, no background music	25
Ease of communication	System to notify at reception or via cards	24
Facility/system improvements	Chairs adjustable for affected side, info sharing, online booking	19
Voice considerations	Loud, slow, clear speech, mask removal	14
General attitude of staff	Understanding and responsive	11
Visual information	Written information, show mouth, speech recognition tools	6
Direction of speech	Speech from better‐hearing side	5
Clear calling system	Visual calling system, reception‐based adjustments	4
Response when not understood	Repeat communication when not understood	3
Others	Pre‐treatment information and support when accompanying children	4

**TABLE 5:** Differences in dental difficulties and accommodation preferences by side of hearing loss.

		Side of hearing loss					
		Left (*n* = 153)			Right (*n* = 220)		
	Items						
Category	Difficulties	No.	%		No.	%	Fisher's P
Before visit	Worried about potential problems due to hearing issues	50	32.7		97	44.1	0.031
	Did not want to visit the clinic because of hearing issues	6	3.9		22	10.0	0.029
	None	65	42.5		69	31.4	0.029
Examination room (other than during treatment)	Had difficulty hearing the dentist explanations and voice	53	34.6		144	65.5	< 0.001
	Had difficulty hearing dental hygienists and assistants	77	50.3		136	61.8	0.033
	Pretended to hear*	56	36.6		114	51.8	0.004
	Interpreted spoken content through imagination	56	36.6		105	47.7	0.034
	None	36	23.5		26	11.8	0.004
During Treatment (In the Examination Room)	Did not notice dentist's voice	17	11.1		51	23.2	0.003
	Could not hear dentist's instructions	41	26.8		124	56.4	< 0.001
	Did not notice assistant's instructions (usually on right side)	37	24.2		23	10.5	< 0.001
	Could not hear assistant's instruction when on right side	63	41.2		39	17.7	< 0.001
	Focused on listening when device sounds stopped	24	15.7		55	25.0	0.039
	Felt greater tension due to hearing difficulty	38	24.8		80	36.4	0.023
	Refrained from speaking out despite anxiety or pain to avoid interrupting treatment	2	1.3		16	7.3	0.007
**Desired action**							
Treatment room response	Sit upright and face patient before speaking	15	9.8		50	22.7	0.001

Shaded cells indicate higher values noted in comparisons between groups.

## References

[1] Y. Agrawal , E. Platz , and J. Niparko , “Prevalence of Hearing Loss and Differences by Demographic Characteristics Among US Adults: Data From the National Health and Nutrition Examination Survey, 1999–2004,” Archives of Internal Medicine 168, no. 14 (2008): 1522–1530. 10.1001/archinte.168.14.1522 18663164

[2] E.‐M. Chia , J. J. Wang , E. Rochtchina , R. R. Cumming , P. Newall , and P. Mitchell , “Hearing Impairment and Health‐Related Quality of Life: The Blue Mountains Hearing Study,” Ear and Hearing 28, no. 2 (2007): 187–195. 10.1097/AUD.0b013e31803126b6 17496670

[3] S. A. Borton , E. Mauze , and J. E. C. Lieu , “Quality of Life in Children With Unilateral Hearing Loss: A Pilot Study,” American Journal of Audiology 19, no. 1 (2010): 61–72. 10.1044/1059-0889(2010/07-0043) PMC3487472 20538966

[4] D. A. Chapman , C. C. Stampfel , J. N. Bodurtha , et al., “Impact of Co‐Occurring Birth Defects on the Timing of Newborn Hearing Screening and Diagnosis,” American Journal of Audiology 20, no. 2 (2011): 132–139. 10.1044/1059-0889(2011/10-0049) PMC4877695 21940980

[5] L. Dalzell , M. Orlando , M. MacDonald et al, “The New York State Universal Newborn Hearing Screening Demonstration Project: Ages of Hearing Loss Identification, Hearing aid Fitting, and Enrollment in Early Intervention,” Ear and Hearing 21, no. 2 (2000): 118–130. 10.1097/00003446-200004000-00006 10777019

[6] D. J. Lee , O. Gómez‐Marín , and H. M. Lee , “Prevalence of Unilateral Hearing Loss in Children: The National Health and Nutrition Examination Survey II and the Hispanic Health and Nutrition Examination Survey,” Ear and Hearing 19 (1998): 329–332. 10.1097/00003446-199808000-00008 9728728

[7] K. J. Cruickshanks , T. L. Wiley , T. S. Tweed , et al., “Prevalence of Hearing Loss in Older Adults in Beaver Dam, Wisconsin. The Epidemiology of Hearing Loss Study,” American Journal of Epidemiology 148, no. 9 (1998): 879–886. 10.1093/oxfordjournals.aje.a009713 9801018

[8] K. Yamamoto , K. Tabei , N. Katsuyama , M. Taira , and K. Kitamura , “Brain Activity in Patients With Unilateral Sensorineural Hearing Loss During Auditory Perception in Noisy Environments,” Journal of Medical and Dental Sciences 64, no. 1 (2017): 19–26. 10.11480/jmds.640103 28367943

[9] L. W. Welsh , J. J. Welsh , L. F. Rosen , and J. E. Dragonette , “Functional Impairments due to Unilateral Deafness,” The Annals of Otology, Rhinology, and Laryngology 113, no. 12 (2004): 987–993. 10.1177/000348940411301209 15633902

[10] R. M. Reeder , J. Cadieux , and J. B. Firszt , “Quantification of Speech‐in‐Noise and Sound Localisation Abilities in Children With Unilateral Hearing Loss and Comparison to Normal Hearing Peers,” Audiology & Neuro‐Otology 20, no. 1 (2015): 31–37. 10.1159/000380745 PMC4467990 25999162

[11] T. Yamada , K. Nozaki , M. Hayashi , and S. Kuwano , “Sound Environment During Dental Treatment in Relation to COVID‐19 Pandemic,” Acoustics 5, no. 4 (2023): 987–998.

[12] J. E. C. Lieu , N. Tye‐Murray , R. K. Karzon , and J. F. Piccirillo , “Unilateral Hearing Loss is Associated With Worse Speech‐Language Scores in Children,” Pediatrics 125, no. 6 (2010): e1348–55. 10.1542/peds.2009-2448 PMC3469199 20457680

[13] Y. Okano , T. Harashima , and A. Katada , “Research on Problems of Hearing and Psychological Aspects in the Daily Life of Persons With Unilateral Hearing Impairment‐use of Social Networking Services,” AUDIOLOGY JAPAN 52, no. 4 (2009): 195–203.

[14] O. M. Cañete , S. C. Purdy , C. R. S. Brown , M. Neeff , and P. R. Thorne , “Behavioural Performance and Self‐Report Measures in Children With Unilateral Hearing Loss due to Congenital Aural Atresia,” Auris, Nasus, Larynx 48, no. 1 (2021): 65–74. 10.1016/j.anl.2020.07.008 32736886

[15] H. A. Snapp and S. A. Ausili , “Hearing With one ear: Consequences and Treatments for Profound Unilateral Hearing Loss,” Journal of Clinical Medicine 9, no. 4 (2020): 1010. 10.3390/jcm9041010 PMC7230949 32260087

[16] V. C. Cannobbio , R. Cartes‐Velásquez , and M. McKee , “Oral Health and Dental Care in Deaf and Hard of Hearing Population: A Scoping Review,” Oral Health & Preventive Dentistry 18, no. 1 (2020): 417–425. 10.3290/j.ohpd.a44687 PMC11654634 32515411

[17] K. Reddy and A. Sharma , “Prevalence of Oral Health Status in Visually Impaired Childre,” Journal of the Indian Society of Pedodontics and Preventive Dentistry 29, no. 1 (2011): 25–27. 10.4103/0970-4388.79922 21521914

[18] A. DeBusschere , M. Lunney , S. Reid , et al., “Hospital Care Does not Meet the Communication Needs of Patients With Hearing Loss: A Qualitative Study of Patient Experiences,” PLoS ONE 20, no. 10 (2025): e0333587. 10.1371/journal.pone.0333587 PMC12513649 41071770

[19] C. Liu , M. M. Mavrommatis , A. Govindan , and M. K. Cosetti , “The Stigma of Hearing Loss: A Scoping Review of the Literature Across Age and Gender,” Otolaryngology–Head and Neck Surgery: Official Journal of American Academy of Otolaryngology‐Head and Neck Surgery 172, no. 6 (2025): 1874–1881. 10.1002/ohn.1246 PMC12120038 40202511

[20] K. Hugdahl , “Fifty Years of Dichotic Listening Research—Still Going and Going and,” Brain and Cognition 76, no. 2 (2011): 211–213. 10.1016/j.bandc.2011.03.006 21470754

[21] D. Kimura , “Some Effects of Temporal‐Lobe Damage on Auditory Perception,” Canadian Journal of Psychology 15, no. 3 (1961): 156–165. 10.1037/h0083218 13756014

[22] T. Watanabe , T. Sugiyama , A. Suzuki , and N. Tamiya , “Communication Difficulties Among Individuals With Hearing Impairments During the COVID‐19 Pandemic and Their Associated Factors: A Cross‐Sectional Study Using a National Survey in Japan,” BMC Public Health [Electronic Resource] 25, no. 1 (2025): 1002. 10.1186/s12889-025-22108-5 PMC11908009 40087703

